# Supplementary material for: Research on the effects of hydrothermal synthesis conditions on the crystal habit of MIL-121
Source: R Soc Open Sci. 2020 Nov 18;7(11):201212. doi: 10.1098/rsos.201212 (PMC7735355; doi:10.1098/rsos.201212)
Supplement: Research on the Effects of Hydrothermal Synthesis Conditions on the Crystal Habit of MIL-121 [file rsos201212supp1.doc]

**ROYAL SOCIETY**

**OPEN SCIENCE**

**Supporting Information**

for R. Soc. open sci.

Research on the Effects of Hydrothermal Synthesis Conditions on the Crystal Habit of MIL-121

**Fei Wang, Liang Zhu*, Qingyan Wei, and Yanfei Wang**

*College of Chemical Engineering and Materials, Tianjin University of Science and Technology Tianjin 300457, China*

**Note S1. SEM Characterizations of the MIL-121 Crystals**

**Figure S1** show the SEM images, PXRD patterns, Raman spectra, FTIR spectra, Raman spectra and Size distribution of five morphologies of MIL-121 crystals synthesized under optimal additive dosage conditions. Figure S1 was SEM images of five morphologies with different magnifications. The difference in the morphology and size of the five MIL-121 crystals can be clearly seen from the images. Figure S1 (a, b) was the SEM image of the crystal obtained under the condition of the optimal solvent addition amount of 40 mL. Figure S1 (c, d) was the SEM image of the crystal obtained under the optimal sodium hydroxide dosage of 8 mmol. Figure S1 (e, f) was the SEM image of the crystal obtained under the optimal lithium chloride dosage of 32 mmol. Figure S1 (g, h) was the SEM image of the crystal obtained under the optimal 2-MI dosage of 8 mmol. Figure S1 (i, j) was the SEM image of the crystal obtained under the condition of raw material ratio of 1:2:2000.

**Note S2. PXRD, FTIR, Raman, Size distribution Characterizations of the MIL-121 Crystals**

**Figure S2** show the PXRD patterns, FTIR spectra, Raman spectra and Size distribution of five morphologies of MIL-121 crystals synthesized under optimal additive dosage conditions. The Figure S2(a) revealed that the PXRD patterns of different morphologies and sizes crystal of MIL-121 were similar to its theoretical PXRD pattern. The peak positions on the FTIR spectra of these five morphologies crystal were similar as shown in Figure S2(b). The Raman spectra of products obtained under different reaction conditions were also identical, which also proved that the product has no change in substance indicated by Figure S2(c). The size distribution diagram of crystal products obtained under different reaction conditions in Figure S2(d), it can be seen that the particle size distribution of the crystal featured by the normal distribution, the particle size distribution range of the product prepared by the addition of solvent amount of 40 mL was 2-40 µm. When the addition amount of sodium hydroxide solution was 8 mmol, the particle size distribution of the product was 2-48 µm. A particle size distribution occurred at the size range from 48 to 70 µm due to larger particles caused by caking after centrifugation and drying. The particle size of the product generated with addition of 32 mmol lithium chloride solution was distributed from 2 to 25 µm. The addition amount of 2-MI approach 8 mmol will produce the product with particle size distribution ranged from 2 to 32 µm. The particles with size distribution more than 32 µm may originated from caking after centrifugation and drying. The results coincided with the experimental phenomenon.

**Note S3. The theoretical PXRD data of MIL-121 crystal**

The theoretical PXRD data (**Table S1**) of MIL-121 crystal was provided by the crystallographic information file ^1^ in the CCDC database.

**Note S4. The particle size distribution data of MIL-121 under different solvent conditions.**

The **Table S2, Table S3, Table S4, Table S5, Table S6** lists the raw data of the particle size distribution test of MIL-121 crystals obtained under the conditions of 10, 20, 30, 40, and 50 mL of solvent.The size of the crystals was characterized using Laser diffraction particle size analyzer (BECKMAN COULTER LS) at a pump speed of 52 and ultrasonic time 5 seconds.

**Note S5. Thermogravimetric analysis of five morphologies of MIL-121**

The **Table S7** lists the changes in the mass loss of the five morphologies of MIL-121 crystals with temperature. It can be seen that at a temperature of 200 °C, the mass loss of the cube morphology obtained under the condition of 40 mL of solvent is 5.5%. The mass loss of the irregular block morphology obtained under the condition of 8 mmol sodium hydroxide is 11.76%. The mass loss of columnar morphology obtained under the condition of 32 mmol of lithium chloride is 5.02%. The mass loss of the octahedral morphology obtained under the condition of 8 mmol of 2-MI is 10%. The mass loss of the stacked sheet morphology obtained under the condition of raw material ratio of 2:1:2000 is 6.31%.


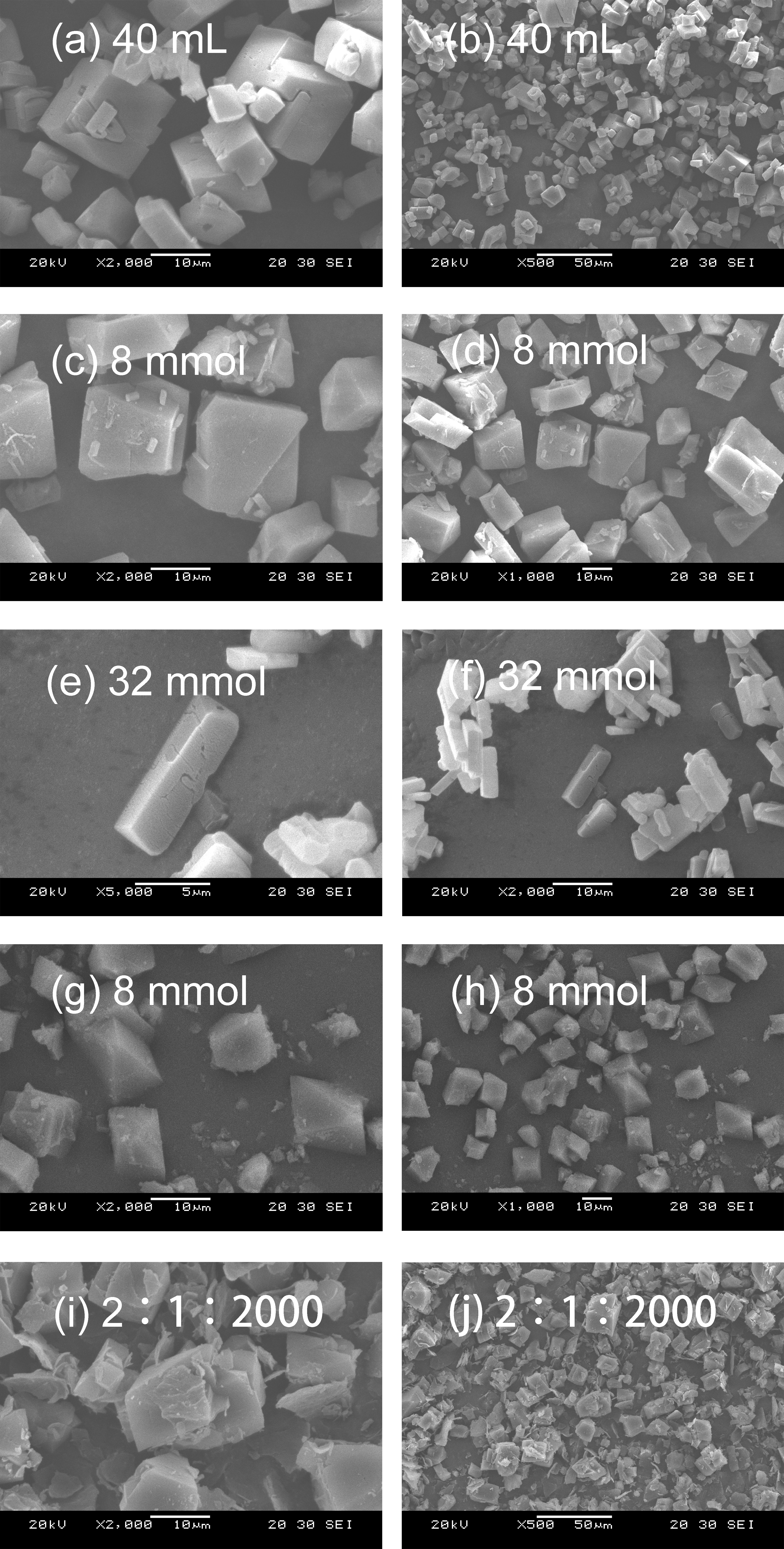


**Figure S1.** The SEM images of MIL-121 prepared at different conditions (a, b) solvent-40 mL, (c, d) NaOH-8 mmol, (e, f) LiCl-32 mmol, (g, h) 2-MI-8 mmol, and (i, j) Al(NO_3_)_3_ ▪9H_2_O∶H_4_BTEC∶H_2_O=2∶1∶2000.

| 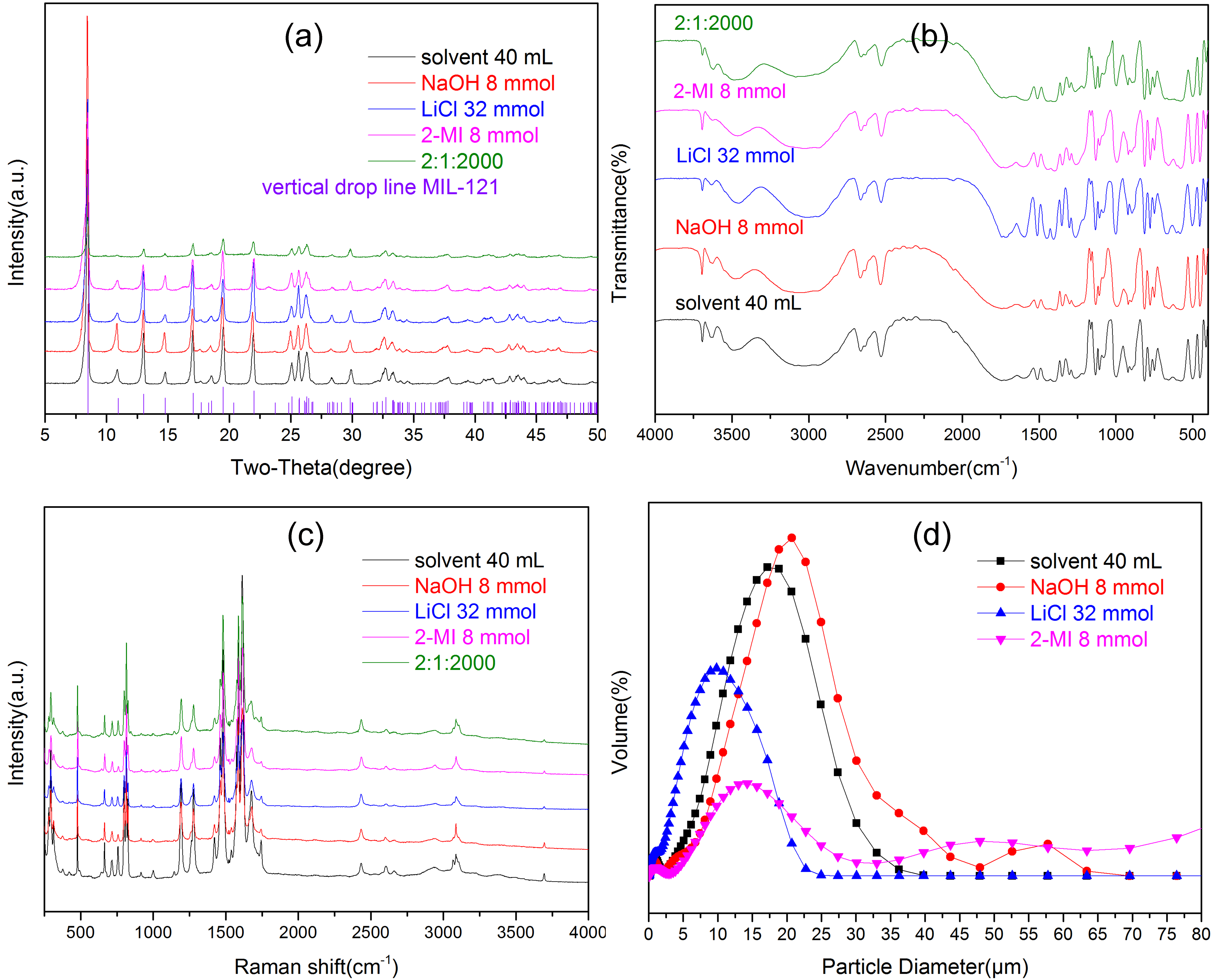  **Figure S2.** The PXRD, FTIR, Raman and size distribution images of MIL-121 synthesized under different temperatures (a) PXRD, (b) FTIR, (c) Raman, (d)Size Distribution. |
| --- |

| 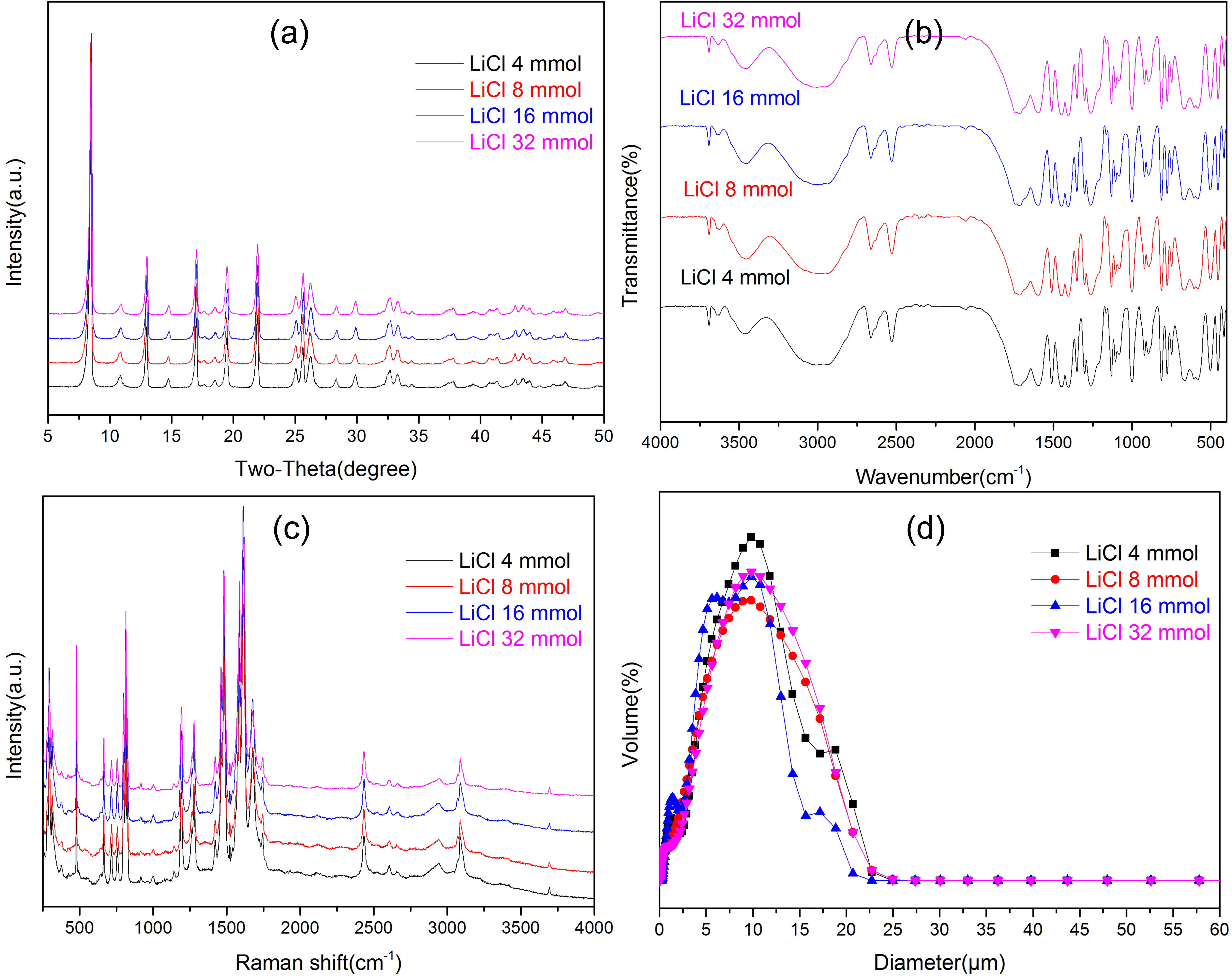  **Figure S3.** The PXRD, FTIR, Raman and size distribution images of MIL-121 prepared at different amount of lithium chloride (a) PXRD, (b) FTIR, (c) Raman, (d) Size Distribution. |
| --- |

**Table S3.** The theoretical PXRD data of MIL-121 crystal.

| 2Theta | d-spacing | Int. | F | h | k | l | Mult. |
| --- | --- | --- | --- | --- | --- | --- | --- |
| 8.506 | 10.3867 | 17303742 | 154.89 | -1 | 1 | 0 | 4 |
| 10.964 | 8.0633 | 763800.2 | 59.44 | 2 | 0 | 0 | 2 |
| 13.03 | 6.7891 | 1518308 | 99.82 | 0 | 2 | 0 | 2 |
| 14.797 | 5.9819 | 815485.5 | 58.87 | -1 | -1 | 1 | 4 |
| 17.06 | 5.1934 | 1669301 | 97.41 | -2 | 2 | 0 | 4 |
| 17.731 | 4.9981 | 19960.15 | 11.08 | -3 | 1 | 0 | 4 |
| 18.331 | 4.836 | 8418.94 | 7.45 | -3 | 1 | 1 | 4 |
| 18.575 | 4.773 | 346039.8 | 48.4 | 1 | -1 | 1 | 4 |
| 19.503 | 4.5479 | 2736715 | 143.13 | 0 | -2 | 1 | 4 |
| 20.363 | 4.3577 | 13591.45 | 10.55 | -1 | 3 | 0 | 4 |
| 22.03 | 4.0316 | 2060231 | 199.26 | 4 | 0 | 0 | 2 |
| 23.746 | 3.744 | 4208.4 | 6.89 | -1 | -3 | 1 | 4 |
| 24.842 | 3.5812 | 16630.81 | 14.35 | -4 | 2 | 1 | 4 |
| 25.117 | 3.5427 | 1084116 | 117.24 | 2 | -2 | 1 | 4 |
| 25.678 | 3.4665 | 581201.3 | 87.86 | -4 | 2 | 0 | 4 |
| 25.71 | 3.4622 | 829072.1 | 105.08 | -3 | 3 | 0 | 4 |
| 26.134 | 3.407 | 334981.5 | 67.96 | -3 | 3 | 1 | 4 |
| 26.232 | 3.3945 | 70432.24 | 44.24 | 0 | 4 | 0 | 2 |
| 26.309 | 3.3847 | 1065470 | 122.05 | 1 | -3 | 1 | 4 |
| 26.469 | 3.3647 | 769165.1 | 104.37 | -5 | 1 | 1 | 4 |
| 26.736 | 3.3317 | 86266.75 | 49.96 | -2 | 0 | 2 | 2 |
| 26.814 | 3.3222 | 134050.9 | 44.17 | 3 | -1 | 1 | 4 |
| 28.022 | 3.1816 | 1231.7 | 4.44 | -3 | -1 | 2 | 4 |
| 28.186 | 3.1635 | 30071.75 | 22.06 | -1 | -1 | 2 | 4 |
| 28.42 | 3.138 | 187118 | 55.51 | -5 | 1 | 0 | 4 |
| 28.507 | 3.1286 | 33147.01 | 23.44 | -2 | 4 | 0 | 4 |
| 28.814 | 3.0959 | 7405.1 | 15.85 | -4 | 0 | 2 | 2 |
| 29.133 | 3.0628 | 43275.3 | 38.77 | 0 | 0 | 2 | 2 |
| 29.849 | 2.991 | 821256.8 | 122.55 | -2 | -2 | 2 | 4 |
| 29.996 | 2.9766 | 21774.29 | 20.06 | -2 | -4 | 1 | 4 |
| 30.073 | 2.9691 | 246.47 | 2.14 | 0 | -4 | 1 | 4 |

**Table S4.** The raw data of the particle size distribution test of MIL-121 obtained under the condition of 10 mL of solvent.

| File name: | Original |  |  |
| --- | --- | --- | --- |
| Operator: | WF |  |  |
| Instrument: | LS 13 320, Aqueous Liquid Module | | |
| Run number: | 534 |  |  |
| Run length: | 64 |  |  |
| Optical model: | Fraunhofer.rf780d PIDS included | | |
| Obscuration: | 11 |  |  |
| PIDS Obscur: | 46 |  |  |
| Obscuration: | OK |  |  |
| Serial Number: | 8321 |  |  |
| From | 0.04 |  |  |
| To | 2000 |  |  |
| Volume | 100 |  |  |
| Mean: | 8.56113 |  |  |
| Median: | 7.96168 |  |  |
| D(0,0): | 0.577741 |  |  |
| Mean/Median ratio: | 1.07529 |  |  |
| Mode: | 11.2932 |  |  |
| S.D.: | 5.42928 |  |  |
| Variance: | 29.4771 |  |  |
| C.V.: | 63.4178 |  |  |
| d10: | 1.4518 |  |  |
| d50: | 7.96168 |  |  |
| d90: | 16.4043 |  |  |

**Table S3.** The raw data of the particle size distribution test of MIL-121 obtained under the condition of 20 mL of solvent.

| File name: | Solvent-20 mL |  |  |
| --- | --- | --- | --- |
| Operator: | WF |  |  |
| Instrument: | LS 13 320, Aqueous Liquid Module | | |
| Run number: | 537 |  |  |
| Run length: | 64 |  |  |
| Optical model: | Fraunhofer.rf780d PIDS included | | |
| Obscuration: | 8 |  |  |
| PIDS Obscur: | 39 |  |  |
| Obscuration: | OK |  |  |
| Serial Number: | 8321 |  |  |
| From | 0.04 |  |  |
| To | 2000 |  |  |
| Volume | 100 |  |  |
| Mean: | 14.1742 |  |  |
| Median: | 13.7933 |  |  |
| D(0,0): | 0.375118 |  |  |
| Mean/Median ratio: | 1.02761 |  |  |
| Mode: | 19.7636 |  |  |
| S.D.: | 7.72693 |  |  |
| Variance: | 59.7054 |  |  |
| C.V.: | 54.5141 |  |  |
| d10: | 4.11178 |  |  |
| d50: | 13.7933 |  |  |
| d90: | 24.0279 |  |  |

**Table S4.** The raw data of the particle size distribution test of MIL-121 obtained under the condition of 30 mL of solvent.

| File name: | Solvent-30 mL |  |  |
| --- | --- | --- | --- |
| Operator: | WF |  |  |
| Instrument: | LS 13 320, Aqueous Liquid Module | | |
| Run number: | 531 |  |  |
| Run length: | 64 |  |  |
| Optical model: | Fraunhofer.rf780d PIDS included | | |
| Obscuration: | 8 |  |  |
| PIDS Obscur: | 37 |  |  |
| Obscuration: | OK |  |  |
| Serial Number: | 8321 |  |  |
| From | 0.04 |  |  |
| To | 2000 |  |  |
| Volume | 100 |  |  |
| Mean: | 13.0907 |  |  |
| Median: | 13.0755 |  |  |
| D(0,0): | 0.0979907 |  |  |
| Mean/Median ratio: | 1.00116 |  |  |
| Mode: | 16.4002 |  |  |
| S.D.: | 7.2749 |  |  |
| Variance: | 52.9242 |  |  |
| C.V.: | 55.5729 |  |  |
| d10: | 1.883 |  |  |
| d50: | 13.0755 |  |  |
| d90: | 22.6383 |  |  |

**Table S5.** The raw data of the particle size distribution test of MIL-121 obtained under the condition of 40 mL of solvent.

| File name: | Solvent-40 mL |  |  |
| --- | --- | --- | --- |
| Operator: | WF |  |  |
| Instrument: | LS 13 320, Aqueous Liquid Module | | |
| Run number: | 528 |  |  |
| Run length: | 64 |  |  |
| Optical model: | Fraunhofer.rf780d PIDS included | | |
| Obscuration: | 8 |  |  |
| PIDS Obscur: | 39 |  |  |
| Obscuration: | OK |  |  |
| Serial Number: | 8321 |  |  |
| From | 0.04 |  |  |
| To | 2000 |  |  |
| Volume | 100 |  |  |
| Mean: | 14.9976 |  |  |
| Median: | 15.1283 |  |  |
| D(0,0): | 0.323866 |  |  |
| Mean/Median ratio: | 0.991361 |  |  |
| Mode: | 18.0035 |  |  |
| S.D.: | 7.3721 |  |  |
| Variance: | 54.3479 |  |  |
| C.V.: | 49.1551 |  |  |
| d10: | 4.76701 |  |  |
| d50: | 15.1283 |  |  |
| d90: | 24.5555 |  |  |

**Table S6.** The raw data of the particle size distribution test of MIL-121 obtained under the condition of 50 mL of solvent.

| File name: | Solvent-50 mL |  |  |
| --- | --- | --- | --- |
| Operator: | WF |  |  |
| Instrument: | LS 13 320, Aqueous Liquid Module | | |
| Run number: | 525 |  |  |
| Run length: | 64 |  |  |
| Optical model: | Fraunhofer.rf780d PIDS included | | |
| Obscuration: | 8 |  |  |
| PIDS Obscur: | 40 |  |  |
| Obscuration: | OK |  |  |
| Serial Number: | 8321 |  |  |
| From | 0.04 |  |  |
| To | 2000 |  |  |
| Volume | 100 |  |  |
| Mean: | 13.0138 |  |  |
| Median: | 13.0445 |  |  |
| D(0,0): | 0.0997216 |  |  |
| Mean/Median ratio: | 0.997643 |  |  |
| Mode: | 16.4002 |  |  |
| S.D.: | 7.26084 |  |  |
| Variance: | 52.7198 |  |  |
| C.V.: | 55.7934 |  |  |
| d10: | 1.62069 |  |  |
| d50: | 13.0445 |  |  |
| d90: | 22.526 |  |  |

**Table S7.** TGA mass loss of five morphologies of MIL-121 crystals synthesized under optimal additive dosage conditions.

| Temperature  ℃ | Weight(%) | | | | |
| --- | --- | --- | --- | --- | --- |
|  | Solvent-40 mL | NaOH-8 mmol | LiCl-32 mmol | 2-MI-8 mmol | 2∶1∶2000 |
| 25 | 0 | 0 | 0 | 0 | 0 |
| 50 | 0.26 | 2.56 | 0.46 | 0.79 | 0 |
| 80 | 0.99 | 5.48 | 1.56 | 3.09 | 0.94 |
| 100 | 1.72 | 7.21 | 2.31 | 5.5 | 2.47 |
| 200 | 5.5 | 11.76 | 5.02 | 10 | 6.31 |
| 250 | 7.07 | 12.76 | 6.31 | 11.32 | 7.56 |
| 280 | 7.65 | 13.06 | 7.06 | 12.05 | 8.29 |
| 300 | 7.74 | 13.15 | 7.19 | 12.53 | 8.45 |
| 320 | 7.84 | 13.27 | 7.32 | 13.1 | 8.64 |
| 350 | 8.09 | 13.52 | 7.63 | 13.81 | 9.05 |
| 380 | 8.54 | 13.8 | 8.31 | 14.66 | 9.74 |
| 400 | 9.13 | 14.11 | 9.32 | 15.41 | 10.54 |
| 420 | 10.16 | 14.69 | 11.09 | 16.4 | 11.81 |
| 450 | 13.67 | 17.13 | 15.92 | 19.36 | 15.81 |
| 480 | 21.37 | 24.02 | 24.58 | 24.9 | 22.79 |
| 500 | 24.7 | 27.16 | 29.08 | 28.49 | 26.97 |
| 520 | 37.15 | 38.45 | 39.74 | 39.35 | 39.1 |
| 550 | 49.4 | 49.07 | 49.69 | 47.53 | 47.14 |
| 580 | 54.48 | 54.18 | 55.12 | 52.92 | 52.62 |
| 600 | 58.02 | 58.09 | 59.02 | 57.15 | 56.6 |
| 620 | 60.62 | 61.02 | 61.82 | 69.47 | 59.71 |
| 650 | 62.65 | 63.26 | 63.94 | 62.99 | 62.12 |
| 680 | 63.38 | 63.99 | 64.67 | 64.12 | 62.87 |
| 720 | 63.85 | 64.45 | 65.16 | 65.09 | 63.32 |
| 780 | 64.4 | 64.97 | 65.71 | 66.24 | 63.86 |

1. C. Volkringer, T. Loiseau, N. Guillou, G. Ferey, M. Haouas, F. Taulelle, E. Elkaim and N. Stock.2010.High-Throughput Aided Synthesis of the Porous Metal-Organic Framework-Type Aluminum Pyromellitate, MIL-121, with Extra Carboxylic Acid Functionalizatio*n.Inorg. Che*m. **4**9, 9852-9862.(10.1021/ic101128w)
